# Changes in Internet Activities and Influencing Factors for Problematic Internet Use During the COVID-19 Pandemic in Korean Adolescents: Repeated Cross-Sectional Study

**DOI:** 10.2196/66448

**Published:** 2025-02-11

**Authors:** Sol I Kim, Jae-Chan Jin, Seo-Koo Yoo, Doug Hyun Han

**Affiliations:** 1 Department of Psychiatry, Woori Children's Hospital Seoul Republic of Korea; 2 School of Social Welfare, Soongsil University Seoul Republic of Korea; 3 Department of Psychiatry, Chung Ang University Hospital Seoul Republic of Korea

**Keywords:** coronavirus pandemic, internet use pattern, internet games, short-form videos, social network system, depressed mood, internet use, pandemic, internet, COVID-19, video, internet behavior, social media, internet addiction, depression, anxiety, digital platforms, mobile phone

## Abstract

**Background:**

As adolescents increasingly engage with digital experiences, the internet serves as a platform for social interaction, entertainment, and learning. The COVID-19 pandemic accelerated this trend, with remote learning and restricted physical interactions driving changes in internet behavior. Adolescents spent more time on gaming and social media, reflecting a notable shift in use patterns.

**Objective:**

We hypothesized that the COVID-19 pandemic changed internet use patterns among Korean adolescents, including content types, time spent on web-based activities, and pathological use prevalence. Additionally, we anticipated that these changes would correlate with shifts in adolescents’ psychological status during the pandemic.

**Methods:**

Data from 827 adolescents aged 12 to 15 years (n=144 in 2018, n=142 in 2019, n=126 in 2020, n=130 in 2021, n=143 in 2022, and n=142 in 2023) were gathered over 6 years from 43 middle schools across 16 regions and 1 hospital in South Korea. The demographic data collected included age, sex, and school year. Participants also provided information on their internet use patterns and levels of internet addiction. Additionally, psychological status, including mood, anxiety, attention, and self-esteem, was assessed.

**Results:**

There were significant differences in the depression scale (Patient Health Questionnaire 9). The Patient Health Questionnaire 9 scores for 2018, 2019, and 2023 decreased compared to those in 2020, 2021, and 2022 (*F*_5_=3.07; *P*=.007). Regarding changes in internet use behavior, game playing among adolescents decreased after the pandemic compared to before, while watching videos increased. Additionally, the rate of problematic internet use was highest for games before COVID-19, but after COVID-19, it was highest for videos, and this trend continued until 2023 (*χ*^2^_3_=8.16, *P*=.04). Furthermore, this study showed that the Young’s Internet Addiction Scale (YIAS) score was highest in the game group in 2018 compared to other groups before COVID-19 (*F*_5_=14.63; *P*<.001). In 2019, both the game and video groups had higher YIAS scores than other groups (*F*_5_=9.37; *P*<.001), and by 2022, the YIAS scores among the game, video, and Social Network Service groups did not differ significantly. The degree of influence on the severity of internet addiction was also greatest for games before COVID-19, but after COVID-19, the effect was greater for videos than for games.

**Conclusions:**

During the COVID-19 pandemic, internet use for academic and commercial purposes, including remote classes and videoconferences, increased rapidly worldwide, leading to a significant rise in overall internet use time. The demand for and dependence on digital platforms is expected to grow even further in the coming era. Until now, concerns have primarily focused on the use of games, but it is now necessary to consider what types of internet behaviors cause problems and how to address them.

## Introduction

### Background

Since its first appearance in a psychiatric context in 1998 [1], the maladaptive and addictive use of the internet, often referred to as internet addiction or internet use disorder, has been increasingly addressed over the past two decades. The internet use rate of Korean adolescents was around 50% in 1999, rising to 99.5% in 2023 [2,3].

Among adolescents, internet applications have shifted from being a supportive function to dominating their daily lives, sometimes leading to addictive use. Both the American Psychiatric Association and the World Health Organization have acknowledged this behavioral phenomenon in their classification manuals, the *Diagnostic and Statistical Manual of Mental Disorders, Fifth Edition* (DSM-5; since 2013 as a condition for further study) and the forthcoming *International Classification of Diseases, 11th Revision* (in the chapter on “Disorders due to behavioral addictions”). However, several authors have criticized this decision, citing a lack of conceptual and empirical foundations [4-9]. Moreover, Griffiths [10] insisted that the context (activities) of internet use is far more important than the amount of time spent on web-based activities.

In almost all studies, adolescents and young adults are consistently identified as the group with the highest prevalence of internet use. As adolescents increasingly engage in digital experiences, the internet serves as a versatile platform for social interaction, entertainment, and information acquisition [11]. Empirical studies have explored the intricate relationships between different types of internet activities and their associated risks. For instance, the problematic use of video games, social media, and the internet has been linked to various mental health issues, such as emotional distress, self-esteem problems, and attention problems, among adolescents [12]. A systematic review of the literature by Coutelle et al [13] suggested that psychological status including inattention, anxiety, and depression significantly impact internet addiction. Additionally, the heterogeneity in internet behavior patterns during the pandemic underscores the importance of considering individual differences when addressing problematic internet use [14].

The COVID-19 pandemic’s impact on internet use highlights the need to understand the complexities of adolescent web-based activities and their mental health. In a review of longitudinal or repeated cross-sectional, follow-up studies, Wolf and Schmitz [15] declared that the COVID-19 pandemic and related stressors could impact the mental health of children and adolescents. Moreover, latent profile analyses reveal distinct patterns of internet and gaming use, suggesting that adolescents’ engagement with digital platforms can be categorized into different profiles based on the intensity and type of use [16-18]. The COVID-19 pandemic further accentuated the use of digital platforms, as social restrictions necessitated remote learning and limited physical interactions. This shift resulted in altered internet behavior patterns among adolescents, with a notable increase in the time spent on internet gaming and social media [11,16].

### Hypothesis

We hypothesized that the COVID-19 pandemic changed psychological status, as well as internet use patterns regarding internet content, use time, and pathological use, in Korean adolescents. Additionally, the severity of pathological internet use was influenced by psychological factors, including mood and attention, during the COVID-19 pandemic.

## Methods

This study is a repeated cross-sectional study that tracked data from 827 students over 6 years.

### Participants

Over 6 years, data from 827 adolescents (n=144 in 2018, n=142 in 2019, n=126 in 2020, n=130 in 2021, n=143 in 2022, and n=142 in 2023) aged 12 to 15 years were gathered from 43 middle schools across 16 regions and 1 hospital in South Korea.

Through a web-based advertisement on the Korean Game Culture Foundation website from January 1, 2018, to December 30, 2023, a total of 69 middle schools from 32 regions and 2 hospitals from 2 regions in South Korea applied to the Visiting Game Class program for game literacy education. Of these, 43 middle schools from 16 regions and 1 hospital were selected through the multistage sampling method. First, the selection was divided by region and hospital; 16 (50%) out of 32 regions were randomly selected, and 1 (50%) of the 2 hospitals in each selected region was chosen at random. Then, among the 52 schools within these 32 regions, 44 (85%) schools were selected. However, 1 school deferred its participation to 2024 due to its academic schedule, so it did not contribute to the research data. Trained agents from the Korean Game Culture Foundation visited schools and hospitals to conduct an investigation.

### Ethical Considerations

All data collected by the agents were anonymized, and participants were rewarded with school supplies worth approximately US $10. Approval for the current study was granted by the Institutional Review Board at Chung-Ang University (1041078-202201-HR-052). We obtained informed consent for research participation from both the students and their parents.

### Demographics and Internet Use Patterns

The demographic data collected included age, sex, and school year. Participants also provided information on their internet use patterns and levels of internet addiction. We defined “problematic internet use” as answering “yes” to the following questions: “Did you hear that people important or close to you consider your internet use to be a problem or suggest you meet a doctor or specialist for it?” [19].

The Young’s Internet Addiction Scale (YIAS), a commonly used instrument for assessing internet addiction and web-based activities such as gaming, was used. This scale comprises 20 items, each rated on a 5-point Likert scale. The internal consistency of the Korean version of the scale has been reported to range from 0.90 to 0.93.

### Psychological Assessment Scales

The Patient Health Questionnaire 9 (PHQ-9) was used to assess depression, with each item rated on a Likert scale from 0 to 3. A cutoff score of 10 (out of 27) was used to indicate depression. Park et al [20] validated the Korean version of the PHQ-9, which demonstrated an internal consistency of α=.81.

The DuPaul Attention-Deficit/Hyperactivity Disorder (ADHD) scale, particularly the ADHD symptom severity scale (ADHD Rating Scale [ARS]), includes 18 items, with 9 items dedicated to inattention and 9 to hyperactivity [21]. So et al [22] validated the Korean version of the ARS (K-ARS) and reported an internal consistency ranging from 0.77 to 0.89.

The Social Phobia Inventory (SPIN) is a self-report questionnaire consisting of 17 items designed to measure three dimensions of social anxiety. Cho et al [23] developed a Korean version of the SPIN (K-SPIN) and reported a high internal consistency with a Cronbach α of 0.91.

The Two-Factor Self-Esteem Scale (SE) is based on a modified version of the Rosenberg Self-Esteem Scale. It conceptualizes self-esteem as an individual’s perception of their worth, incorporating elements of self-respect and self-confidence [24]. This scale contains 10 statements that assess overall feelings toward oneself. Participants indicate their level of agreement on a 4-point Likert scale, ranging from 1 (disagree completely) to 4 (agree completely). The internal consistency of the Korean version of the scale, referred to as the Self-Esteem Scale-Korean, has been reported with a Cronbach α of 0.79 [25].

### Data Analysis

Demographic characteristics, including age, school year, and internet use time across years, were analyzed using ANOVA tests. Sex and internet activity across years were analyzed using chi-square tests. The YIAS scores and psychological scale scores, including PHQ-9, K-ARS, K-SPIN, and SE were also analyzed using ANOVA tests. The correlations between age, SE, PHQ-9, K-SPIN, K-ARS, IT use time, and YIAS were assessed using Pearson correlation analyses. The correlations between sex (IT activity) and age, SE, PHQ-9, K-SPIN, K-ARS, IT use time, and YIAS were assessed using Spearman correlation analyses. The correlation between sex and IT activity was assessed using Kendall tau-b correlation analysis.

We conducted hierarchical linear regression analyses using YIAS scores as the dependent variable to identify factors influencing the severity of problematic internet use. In Model 1, we tested the associations of demographic factors with the severity of problematic internet use. In Model 2, psychological factors were added to test their associations beyond the effects of demographic factors. In Model 3, internet use time was added to test its association beyond the effects of demographic and psychological factors. Finally, in Model 4, internet activities were added to test their associations beyond the effects of demographic factors, psychological factors, and internet use time. Statistical significance was set a priori at α=.05 (two-sided) to limit type-I error. All analyses were conducted using the *Complex Samples* module of the *PASW* statistics software package (version 19; IBM Corp).

## Results

### Demographic and Clinical Characteristics

There were no differences in sex ratio and age across the 6 years. Similarly, there were no differences in the scores of the self-esteem, social anxiety, and attention scales over the same period. However, significant differences were observed in the PHQ-9 scores (*F*_5_=3.07; *P*=.007). The PHQ-9 scores for 2018, 2019, and 2023 were lower compared to those in 2020, 2021, and 2022. Additionally, there was a significant difference in internet use time (*F*_5_=6.30; *P*<.001). Internet use time was highest in 2020 and 2021, followed by 2022 and 2023, and lowest in 2018 and 2019 (Table 1).

**Table 1:** Demographic characteristics.

				2018 (n=144)		2019 (n=142)		2020 (n=126)		2021 (n=130)		2022 (n=143)		2023 (n=142)
**Demographic characteristics**														
	**Sex, n (%)**													
		Male	83 (57.6)		80 (56.3)		74 (58.7)		77 (59.2)		80 (55.9)		86 (60.6)	
		Female	61 (42.4)		62 (43.7)		52 (41.3)		53 (40.8)		63 (44.1)		56 (39.4)	
	**Age (years), mean** **(SD)**			13.56 (1.10)		13.59 (0.59)		13.55 (1.11)		13.34 (1.33)		13.35 (0.73)		13.64 (0.92)
**IT use pattern**														
	Internet use time^a^, mean (SD)			2.82 (1.23)		3.09 (1.67)		3.55 (1.14)		3.69 (1.78)		3.21 (1.01)		3.20 (1.71)
	YIAS^b^, mean (SD)			45.08 (15.67)		43.87 (12.83)		43.87 (13.17)		45.08 (13.81)		44.41 (13.05)		44.32 (14.61)
**Psychological scales**														
	SE^c^, mean (SD)			27.09 (5.09)		27.20 (3.13)		27.33 (7.16)		28.15 (5.33)		27.89 (5.02)		27.04 (3.21)
	PHQ-9^d,e^, mean (SD)			9.69 (7.76)		9.88 (5.24)		11.90 (4.66)		11.13 (6.84)		11.13 (5.93)		9.58 (7.14)
	K-SPIN^f^, mean (SD)			18.58 (11.05)		18.65 (11.92)		19.61 (11.51)		20.11 (12.75)		19.39 (13.51)		18.37 (12.89)
	K-ARS^g^, mean (SD)			10.37 (7.98)		9.19 (8.82)		9.17 (8.09)		10.01 (9.37)		10.10 (9.96)		9.28 (9.30)

^a^*F*_5_=6.30; *P*<.001; 2018=2019<2022=2023<2020=2021.

^b^YIAS: Young’s Internet Addiction Scale.

^c^SE: Two-Factor Self-Esteem Scale.

^d^PHQ-9: Patient Health Questionnaire 9.

^e^*F*_5_=3.07; *P*=.007; 2018=2019=2023<2020=2021=2022.

^f^K-SPIN: Korean version of the Social Phobia Inventory.

^g^K-ARS: Korean version of the Attention Deficit/Hyperactivity Disorder Rating Scale.

### The Correlations Between All Variables

In the comparison of variable correlations, PHQ-9 scores were positively correlated with K-ARS scores (*r*=0.45; *P*<.001). YIAS scores were positively correlated with PHQ-9 scores (*r*=0.43; *P*<.001) and K-ARS scores (*r*=0.43; *P*<.001; Table 2).

**Table 2:** Correlation matrix of all variables^a^.

Variables		Age	Sex	SE^b^	PHQ-9^c^	K-SPIN^d^	K-ARS^e^	IT use time	IT activity	YIAS^f^
**Age**										
	*r*	1	–0.05	–0.04	0.04	0.07	0.10	0.09	0.09	0.05
	*P* value	—^g^	.11	.25	.31	.06	.004	.007	.03	.09
**Sex**										
	*r*	–0.05	1	–0.06	–0.01	0.05	–0.32	0.02	0.15	–0.07
	*P* value	.11	—	.12	.55	.19	.75	.53	<.001	.03
**SE**										
	*r*	–0.04	–0.06	1	–0.03	–0.21	–0.13	–0.02	–0.04	–0.02
	*P* value	.25	.12	—	.38	<.001	<.001	.49	.21	.25
**PHQ-9**										
	*r*	0.04	–0.03	–0.03	1	0.14	0.45	0.07	0.08	0.43
	*P* value	.31	.55	.38	—	<.001	<.001	.05	.02	<.001
**K-SPIN**										
	*r*	0.07	0.05	–0.21	0.14	1	0.28	0.05	0.06	0.18
	*P* value	.06	.19	<.001	<.001	—	<.001	.16	.08	<.001
**K-ARS**										
	*r*	0.10	–0.32	–0.13	0.45	0.28	1	0.08	0.04	0.43
	*P* value	.004	.75	<.001	<.001	<.001	—	.03	.29	<.001
**IT use time**										
	*r*	0.09	0.02	–0.02	0.07	0.05	0.08	1	0.52	0.08
	*P* value	.007	.53	.49	.05	.16	.03	—	.14	.04
**IT activity**										
	*r*	0.09	0.15	–0.04	0.08	0.06	0.04	0.52	1	0.03
	*P* value	.03	<.001	.21	.02	.08	.29	.14	—	.34
**YIAS**										
	*r*	0.05	–0.07	–0.02	0.43	0.18	0.43	0.08	0.03	1
	*P* value	.09	.03	.25	<.001	<.001	<.001	.04	.34	—

^a^Pearson correlation: age, SE, PHQ-9, K-SPIN, K-ARS, IT use time, YIAS; Spearman correlation: sex, IT activity versus age, SE, PHQ-9, K-SPIN, K-ARS, IT use time, YIAS; Kendall tau-b: sex, IT activity.

^b^SE: Two-Factor Self-Esteem Scale.

^c^PHQ-9: Patient Health Questionnaire 9.

^d^K-SPIN: Korean version of the Social Phobia Inventory.

^e^K-ARS: Korean version of the Attention Deficit Hyperactivity Disorder Scale.

^f^YIAS: Young’s Internet Addiction Scale score.

^g^Not applicable.

### Changes in Internet Activities of Korean Adolescents Over 6 Years

**Figure 1:**
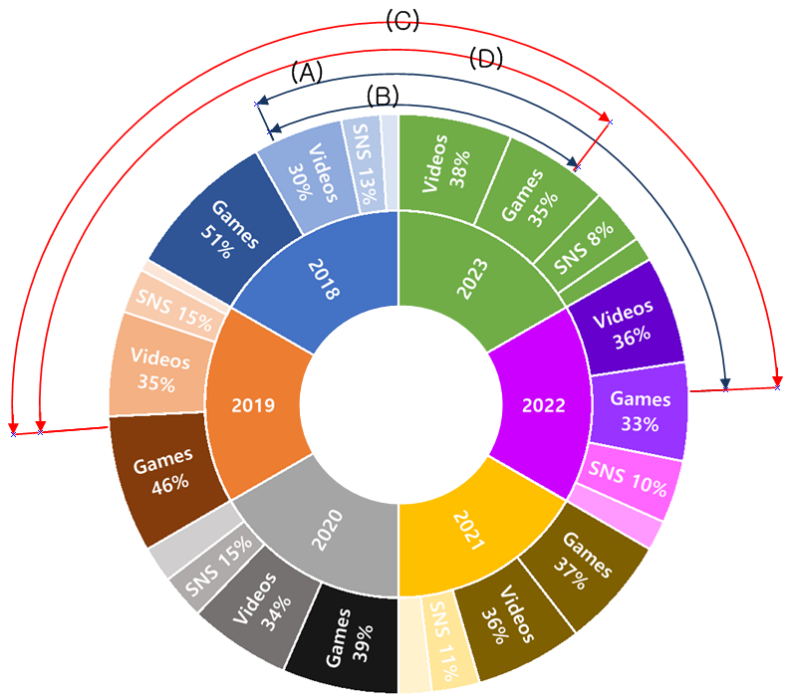
Changes in internet activities of Korean adolescents over 6 years (chi-square test). (A) Comparison of internet activities between 2018 and 2022 (*χ*^2^_3_=11.20, *P*=.01); (B) comparison of internet activities between 2018 and 2023 (*χ*^2^_3_=12.32, *P*=.006); (C) comparison of internet activities between 2019 and 2022 (*χ*^2^_3_=8.68, *P*=.03); and (D) comparison of internet activities between 2019 and 2023 (*χ*^2^_3_=8.16, *P*=.04). SNS: Social Network Service.

Over the past 6 years, the population engaged in gameplay had decreased, while the population watching videos had increased. In 2018, a total of 51.4% (74/144) of the population engaged in gameplay, and 29.9% (43/144) watched videos. By 2022, these figures had shifted to 32.9% (47/143) for gameplay and 35.7% (51/143) for video watching, and in 2023, to 35% (50/142) for gameplay and 37.8% (54/142) for video watching (2022: *χ*^2^_3_=11.20, *P*=.01; 2023: *χ*^2^_3_=12.32, *P*=.006). Similarly, in 2019, a total of 46.2% (66/142) engaged in gameplay and 35% (50/142) watched videos. By 2022, these figures had changed to 32.9% (47/143) for gameplay and 35.7% (51/143) for video watching, and in 2023, to 35% (50/142) for gameplay and 37.8% (54/142) for video watching (2022: *χ*^2^_3_=8.68, *P*=.03; 2023: *χ*^2^_3_=8.16, *P*=.04; Figure 1).

### Differences in Problematic Internet Use for 4 Activities in Korean Adolescents Over 6 Years

**Figure 2:**
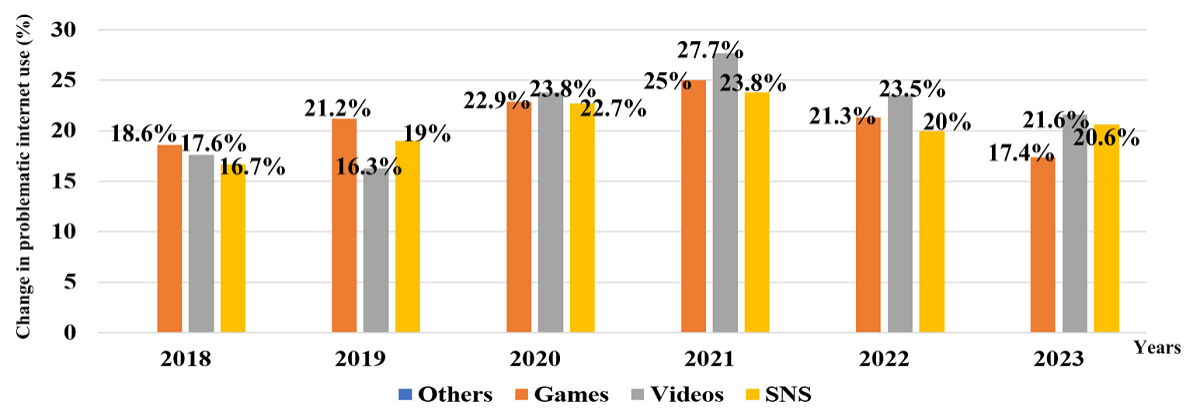
Changes in problematic internet use for 4 activities in Korean adolescents over 6 years. SNS: Social Network Service.

Until 2019, the proportion of problematic internet use was highest in the gaming group among the 4 types of internet use. However, the proportion of problematic internet use in watching videos abruptly increased in 2020 and has maintained its top position until 2023. The proportion of problematic internet use in Social Network Service (SNS) use continuously increased until 2021 but decreased in 2022 and 2023 (Figure 2).

### Differences in YIAS Scores for 4 Activities in Korean Adolescents Over 6 Years

**Figure 3:**
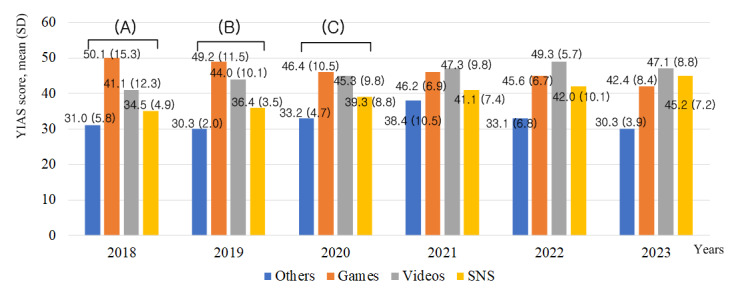
The changes in YIAS scores for 4 activities in Korean adolescents over 6 years. (A) Game>Videos>SNS=Others (*F*_3_=14.63; *P*<.001). (B) Game=Videos>SNS=Others (*F*_3_=9.37; *P*<.001). (C): Game=Videos>SNS=Others (*F*_3_=4.98; *P*=.003). SNS: Social Network Service; YIAS: Young’s Internet Addiction Scale.

In 2018, the YIAS scores for gameplay (mean 50.1, SD 15.3) were the highest compared to other activities. The YIAS scores for watching videos (mean 41.1, SD 12.3) were higher than those for SNS use (mean 34.5, SD 4.9) and other activities (mean 31.0, SD 5.8; *F*_3_=14.63; *P*<.001). In 2019, the YIAS scores for gameplay (mean 49.2, SD 11.5) and watching videos (mean 44.0, SD 10.1) were higher than those for SNS use (mean 36.4, SD 3.5) and other activities (mean 30.3, SD 2.0; *F*_3_=9.37; *P*<.001). In 2020, the YIAS scores for gameplay (mean 46.4, SD 10.5) and watching videos (mean 45.3, SD 9.8) were higher than those for SNS use (mean 39.3, SD 8.8) and other activities (mean 33.2, SD 4.7; *F*_3_=4.98; *P*=.003). In 2021, there was no significant difference between the scores of the four activities (*F*_3_=2.33; *P*=.08). In 2022, the YIAS scores for other activities (mean 33.1, SD 6.8) were significantly lower than those for gameplay (mean 45.6, SD 6.7), watching videos (mean 49.3, SD 5.7), and SNS use (mean 42.0, SD 10.1; *F*_3_=6.83; *P*<.001). However, there was no significant difference between the scores for gameplay, watching videos, and SNS use. In 2023, the YIAS scores for other activities (mean 30.3, SD 3.9) were significantly lower, than those for gameplay (mean 42.4, SD 8.4), watching videos (mean 47.1, SD 8.8), and SNS use (mean 45.2, SD 7.2; *F*_3_=4.73; *P*=.004). However, there was no significant difference between the scores for gameplay, watching videos, and SNS use (Figure 3).

### Differences in Influencing Factors for YIAS Scores

Considering the beta values of Model 4, the order of statistically significant influences on the severity of internet addiction in 2018 was as follows: game playing, PHQ-9 scores, watching videos, K-ARS scores, and IT use time (Table 3 and Table S1 in Multimedia Appendix 1). In 2019, the order was game playing, watching videos, PHQ-9 scores, K-ARS scores, and IT use time (Table 3 and Table S2 in Multimedia Appendix 1). In 2020, the order was K-ARS scores, game playing, and watching videos (Table 3 and Table S3 in Multimedia Appendix 1). In 2021, the order was PHQ-9 scores and watching videos (Table 3 and Table S4 in Multimedia Appendix 1). In 2022, the order was watching videos, game playing, PHQ-9 scores, K-ARS scores, and SNS use (Table 3 and Table S5 in Multimedia Appendix 1). In 2023, the order was PHQ-9 scores, K-ARS scores, watching videos, SNS use, and game playing (Table 3 and Table S6 in Multimedia Appendix 1).

**Table 3:** Differences in influencing factors for YIAS^a^ scores over 6 years.

Variables			2018		2019		2020		2021		2022		2023
**Demographic factors**													
	Age	—^b^		—		—		—		—		—	
	Sex	—		—		—		—		—		—	
**Psychological test**													
	SE^c^	—		—		—		—		—		—	
	PHQ-9^d^	X2^e^ (0.40)		X3 (0.30)		—		X1 (0.51)		X3 (0.31)		X1 (0.57)	
	K-SPIN^f^	—		—		—		—		—		—	
	K-ARS^g^	X4 (0.23)		X4 (0.30)		X1 (0.60)		—		X4 (0.25)		X2 (0.33)	
IT use time			X5 (0.17)		X5 (0.13)		—		—		—		—
**IT activity**													
	Game	X1 (0.52)		X1 (0.39)		X2 (0.24)		—		X2 (0.37)		X5 (0.24)	
	Videos	X3 (0.26)		X2 (0.36)		X3 (0.24)		X2 (0.25)		X1 (0.43)		X3 (0.28)	
	SNS^h^	—		—		—		—		X5 (0.25)		X4 (0.26)	

^a^YIAS: Young’s Internet Addiction Scale.

^b^Not applicable.

^c^SE: Two-Factor Self-Esteem Scale.

^d^PHQ-9: Patient Health Questionnaire 9.

^e^Xn (beta value): X: statistically significant; n: ranking; 1: most effective factor.

^f^K-SPIN: Korean version of the Social Phobia Inventory.

^g^K-ARS: Korean version of the Attention Deficit/Hyperactivity Disorder Rating Scale.

^h^SNS: Social Network Service.

## Discussion

### Principal Findings

The goal of this study was to examine changes in internet use patterns among adolescents, from before to after the outbreak of the COVID-19 pandemic. Our findings revealed several changes in internet use time and patterns, as well as shifts in problematic use behavior before, during, and after COVID-19.

The results showed that PHQ-9 scores were low before and after the COVID-19 pandemic, but high from 2020 to 2022, when the pandemic was at its peak. This aligns with previous studies indicating a high proportion of adolescents experiencing depression and anxiety during the pandemic [26,27]. The COVID-19 pandemic brought significant changes to adolescents’ lives, potentially acting as environmental stressors [28]. To avoid exposure to the virus, young people actively avoided social activities, and many children and adolescents were confined to their homes for extended periods due to lockdowns. This social isolation has been associated with an increased risk of depression and anxiety in children and adolescents [29]. Additionally, the fear of infection itself was linked to anxiety and depression [30]. With the lifting of lockdowns, most people have resumed their lives, and depression and anxiety likely diminished since the peak of the pandemic due to the development of coping mechanisms and hopeful news about vaccines during the “honeymoon phase” of the disaster [31-33].

Similar results regarding the relationship between mental health and internet addiction during the COVID-19 pandemic were reported in several studies [34,35]. Ye et al [34] reported that depression is positively correlated with internet addiction during the COVID-19 pandemic. Moreover, adolescents with depressive disorders could have a higher risk of internet addiction. In a meta-analysis, Tang et al [35] reported that the association between problematic smartphone use and depressive symptoms became stronger after the COVID-19 outbreak.

To our knowledge, there is evidence of changes in internet use patterns during the pandemic, including increased dependence on the internet [36]. In a large, national youth sample, cross-sectional study conducted in the United States during the early period of the COVID-19 pandemic, the absolute time of internet use among teenagers more than doubled compared to prepandemic times [37]. Additionally, a systematic review and meta-analysis of screen time among children and youth aged 0 to 21 years before and after the pandemic showed a 1.6-fold increase in screen time during the pandemic [38]. Similarly, in this study, from 2020 to 2021, during the COVID-19 outbreak, adolescents used the internet more than before, and their internet use time decreased as the COVID-19 peak passed. Previous studies have shown that internet use time increased as physical activity decreased due to the lockdowns caused by COVID-19 [39,40]. These results may reflect decreased screen time as physical and offline activity increased when daily life recovered after COVID-19 [41].

During the pandemic, teenagers increased their internet use for various purposes, such as interacting with friends, doing homework, enjoying games, and attending remote classes [42]. Generally, internet addiction is suspected when an individual devotes excessive time to internet use [43]. Excessive internet use is known to likely lead to internet addiction, especially in children and adolescents [44]. Similarly, as adolescents spend more time on web-based activities during the pandemic, many studies have shown an increased risk of internet addiction. For instance, a study examining internet addiction in Taiwanese high school students during COVID-19 found a 24.4% rate of addiction, indicating an increase compared to prepandemic levels [45]. In a longitudinal study on the developmental qualities of children and adolescents during the COVID-19 pandemic, Wang et al [46] suggested that the pandemic may lead to a decline in positive youth development, making them more vulnerable to internet addiction. Additionally, a study conducted in India investigated the effect of the COVID-19 lockdown on internet addiction in late teenagers, showing a 14.84% increase in internet gaming disorder (IGD) frequency compared to previous studies in the same region [47]. However, most studies focused only on excessive internet use and did not differentiate specific internet activities. Therefore, to address problematic internet use among adolescents, it is necessary to examine in more detail which specific internet activities are problematic.

Looking at the changes in internet use behavior that this study focused on, game playing decreased in adolescents after the pandemic compared to before the pandemic, and watching videos increased further. In addition, in this paper, the problematic internet use rate of games was the highest before COVID-19. Still, after COVID-19, the problematic internet use rate was the highest in the video group, and this trend continued until 2023. In addition, as our study showed, the YIAS score was the highest in the game group in 2018 compared to other groups before COVID-19. Still, in 2019, the YIAS scores in the game and video groups were higher than other groups, and the YIAS scores between the game, video, and SNS groups did not differ significantly as we went into 2022. The degree of influence on the severity of internet addiction was also the largest in the game group before COVID-19, but the effect on the video group was greater than in the game group after COVID-19.

Similar to these findings, several studies have reported significant changes in how individuals allocate time across different activities during the COVID-19 pandemic, noting increased SNS use, watching videos, and more, not just gaming [36,48,49]. According to a probability-based tracking survey of tweens and teens in the United States, there was no significant difference in teen gaming time between 2019 and 2021. Still, the time spent watching videos increased significantly, up to 23 minutes daily [50]. As several reports suggest, overall, the global gaming market has shrunk since the COVID-19 pandemic [51], and gaming users’ gaming hours have declined since the peak [52]. On the other hand, the video-related industry has grown significantly as lockdowns have made it one of the major recreational activities [53]. According to Morse et al [54], TV or streams or movies have emerged as a new leisure activity, with activities experiencing the greatest increase during COVID-19. Another study suggests that Netflix, Hulu, and Amazon Prime Video are now recognized as some of the most important TV networks and video sources for the younger adult generation, further highlighting the popularity of streaming services [55]. Similarly, other studies point to the problematic use of SNS platforms, including video consumption by adolescents [36,56,57].

In the paper by Nawaz et al [36] on technology utilization in the new post–COVID-19 era, social networking platform engagement has increased markedly as study participants have been given more time for web-based social interaction. Meanwhile, in one study conducted in Italy, video consumption through certain platforms, like TikTok, during the COVID-19 pandemic strongly predicted social media addiction [56]. In addition, similar to previous studies that revealed that adolescents’ social anxiety can lead to problematic social media use [57]; the increasing web-based social interaction trend, rather than internet gaming, reflects the growing dependence on digital connections as a coping mechanism during physical distancing and quarantines, with web-based streaming services, including videos, also experiencing notable use increases [36]. Taken together, even before COVID-19, video-sharing platforms such as YouTube [58] and social media platforms such as Instagram or Snapchat were already gaining popularity [59]. However, short video consumption worldwide saw the fastest and largest increase in the early stages of the pandemic, especially among those aged 15-29 years [60], where teenagers would have sought self-expression and social rewards by recording and communicating their daily lives [61]. Furthermore, the rise in the use of SNS and video apps during COVID-19 suggests a shift in overall trends during physical distancing, searching for information, and using platforms to help maintain social relationships [62].

As mentioned in the previous results, the COVID-19 pandemic has significantly changed people’s lives worldwide, with internet use at the center of this change. Although internet use has alleviated mental health symptoms for many and helped them cope with new trends [63,64], adolescents have been able to devote more time to web-based activities, especially during emotionally demanding times, which can lead to problematic use [65]. It is essential to provide guidance to reduce the risk of such addiction. Adults should observe how much time adolescents spend on web-based activities (eg, playing games and watching videos) and help them manage these activities [48].

However, problematic internet use has not yet been sufficiently discussed. The American Psychiatric Association included IGD in *DSM-5* [66], and the World Health Organization included gaming disorder in the *International Classification of Diseases, 11th Revision* [67], but current societies present only diagnostic criteria for games. This study shows that games no longer account for a high percentage of adolescents’ problematic internet use time. In addition, the *DSM-5* acknowledges the limitations of the absence of well-studied subtypes for IGD and acknowledges that there are limitations to the diagnosis, such as the fact that it is not clear which game type is specifically included in IGD diagnosis [66]. Furthermore, a survey of adolescents in China on IGD, problematic smartphone use, and problematic SNS use found that each has a different core symptom, with problematic SNS use requiring a different therapeutic approach as it shows a different core symptom [68]. Similarly, as Griffiths [10] argued in his study on the concept of internet addiction and IGD, it should be understood that people addicted to web-based activities, such as web-based games, web-based gambling, web-based sex, and web-based shopping, should not be defined as people with internet addictions, but rather as people with game addictions, sex addictions, or shopping addictions, who are engaged in addictive behavior using the internet as a tool. Chen et al [69] reported that problematic smartphone use was associated with the COVID-19 pandemic outbreak, whereas problematic internet gaming was not. Previous studies suggest that the focus should be on how the internet is used, rather than seeing the excessive internet use itself as the problem. It is not just gameplay time that is a problem—as individual internet behavior patterns have changed during the COVID-19 pandemic, these points should be considered when solving problematic internet use.

### Limitations

There are some limitations to this study. First, there may be sampling errors in representing the overall internet use patterns of teenagers, as the survey only included teenagers aged 13 to 15 years. Second, because this study did not track the same population over 6 years, it was unable to fully capture the trends in internet addiction and patterns of internet use among adolescents. Third, this study could not fully capture changes in internet addiction and mental health because it was not a longitudinal study within a single group.

Although literature on the pandemic has surged with the global spread of COVID-19, little has been studied about the changes in media and content use caused by the pandemic [70]. To the best of our knowledge, this is the first study to focus on changes in internet use patterns due to the pandemic. The strength of our study lies in surveying the same teenage group over 6 years before, during, and after COVID-19 and further investigating which types of internet use were identified as problematic. Based on these changes in internet use patterns and problems among teenagers, this study contributes to the literature on understanding the trends in internet use behavior caused by COVID-19 and helps predict future changes in internet use.

### Conclusions

During the COVID-19 pandemic, academic and commercial internet use through remote classes and videoconferences increased rapidly worldwide, leading to a rise in overall internet use time. The demand for digital platforms will continue to grow in the coming era. Until now, discussions have primarily focused on the use of games, but it is now necessary to consider what types of internet behavior cause problems and how to address them.

## Appendix

Multimedia Appendix 1 Hierarchical linear regression analyses.

## Abbreviations

ADHD: attention-deficit/hyperactivity disorder
ARS: Attention-Deficit/Hyperactivity Disorder Rating Scale
DSM-5: Diagnostic and Statistical Manual of Mental Disorders, Fifth Edition
IGD: internet gaming disorder
K-ARS: Korean version of the Attention-Deficit/Hyperactivity Disorder Rating Scale
K-SPIN: Korean version of the Social Phobia Inventory
PHQ-9: Patient Health Questionnaire 9
SE: Two-Factor Self-Esteem Scale
SNS: Social Network Service
SPIN: Social Phobia Inventory
YIAS: Young’s Internet Addiction Scale

## Data Availability

The datasets generated and analyzed during this study are not publicly available as they contain information that could compromise the privacy and consent of the research participants but are available from the corresponding author upon reasonable request.
