# Supplementary material for: Changes in Internet Activities and Influencing Factors for Problematic Internet Use During the COVID-19 Pandemic in Korean Adolescents: Repeated Cross-Sectional Study
Source: JMIR Pediatr Parent. 2025 Feb 11;8:e66448. doi: 10.2196/66448 (PMC11862775; doi:10.2196/66448)
Supplement: Multimedia Appendix 1 [file pediatrics_v8i1e66448_app1.docx]

Table S1. Hierarchical linear regression analyses with the YIAS score as the dependent variable in 2018

| Independent variables | | Model 1 | | | Model 2 | | | Model 3 | | | Model 4 | | |
| --- | --- | --- | --- | --- | --- | --- | --- | --- | --- | --- | --- | --- | --- |
|  |  | B | Beta | t | B | Beta | t | B | Beta | t | B | Beta | t |
| Demographic factors | Age | -5.06 | -0.16 | -1.86 | -4.74 | -0.15 | -2.04 | -4.51 | -0.14 | -1.95 | -3.26 | -0.10 | -1.57 |
|  | Sex (female) | -0.23 | -0.02 | -0.19 | -1.26 | -0.09 | -1.21 | -0.68 | -0.05 | -0.64 | 0.08 | 0.01 | 0.09 |
| Psychological  Test | SE |  |  |  | -0.03 | -0.02 | -0.35 | -0.04 | -0.03 | -0.44 | 0.00 | 0.00 | -0.01 |
|  | PHQ9 |  |  |  | 0.73 | 0.36 | 4.70^***^ | 0.72 | 0.36 | 4.64^***^ | 0.81 | 0.40 | 5.81^***^ |
|  | KSPIN |  |  |  | -0.02 | -0.01 | -0.08 | -0.01 | 0.00 | -0.02 | -0.07 | -0.02 | -0.36 |
|  | KARS |  |  |  | 0.61 | 0.31 | 4.05^***^ | 0.65 | 0.33 | 4.31^***^ | 0.45 | 0.23 | 3.26^**^ |
| IT use time | Time |  |  |  |  |  |  | -1.77 | -0.14 | -1.90 | 2.18 | -0.17 | -2.54^*^ |
| IT activity | Game |  |  |  |  |  |  |  |  |  | 16.31 | 0.52 | 3.92^**^ |
|  | Videos |  |  |  |  |  |  |  |  |  | 8.84 | 0.26 | 2.00^*^ |
|  | SNS |  |  |  |  |  |  |  |  |  | -0.24 | -0.01 | -0.05 |
| Statistics of the model | | F = 1.981, R^2^ = .027 | | | F = 12.210^***^, R^2^ = .348,  F Change = 16.879^***^,  R^2^ Change = 0.321 | | | F = 11.182^**^, R^2^ = .365,  F Change = 3.612,  R^2^ Change =0.017 | | | F = 13.905^**^, R^2^ = .511,  F Change = 13.225^***^,  R^2^ Change = 0.146 | | |

*: *P*<.05, **: *P*<.01, ***: *P*<.001, YIAS: Young Internet Addiction Scale Score, SE: Two-Factor Self-Esteem Scale, PHQ-9: The Patient Health Questionnaire 9, K-SPIN: The Korean version of the Social Phobia Inventory, K-ARS: Korean version of the attention deficit hyperactivity disorder scale

Table S2. Hierarchical linear regression analyses with the YIAS score as the dependent variable in 2019

| Independent variables | | Model 1 | | | Model 2 | | | Model 3 | | | Model 4 | | |
| --- | --- | --- | --- | --- | --- | --- | --- | --- | --- | --- | --- | --- | --- |
|  |  | B | Beta | t | B | Beta | t | B | Beta | t | B | Beta | t |
| Demographic factors | Age | -2.60 | -0.10 | -1.17 | -2.84 | -0.11 | -1.56 | -3.01 | -0.12 | -1.69 | -1.871 | -0.073 | -1.08 |
|  | Sex (female) | 3.83 | 0.18 | 2.07^*^ | 2.32 | 0.11 | 1.52 | 1.50 | 0.07 | 0.98 | 1.03 | 0.05 | 0.70 |
| Psychological  Test | SE |  |  |  | 0.20 | 0.18 | 2.55^*^ | 0.20 | 0.18 | 2.60^*^ | 0.18 | 0.16 | 2.46^*^ |
|  | PHQ9 |  |  |  | 0.64 | 0.26 | 3.48^**^ | 0.63 | 0.26 | 3.51^**^ | 0.74 | 0.30 | 4.17^**^ |
|  | KSPIN |  |  |  | 0.30 | 0.07 | 1.04 | 0.26 | 0.06 | 0.93 | 0.16 | 0.04 | 0.61 |
|  | KARS |  |  |  | 0.47 | 0.32 | 4.07^***^ | 0.45 | 0.31 | 3.97^***^ | 0.436 | 0.30 | 4.03^***^ |
| IT use time | Time |  |  |  |  |  |  | 1.36 | 0.18 | 2.60^*^ | 0.98 | 0.13 | 1.95^*^ |
| IT activity | Game |  |  |  |  |  |  |  |  |  | 9.90 | 0.39 | 2.37^*^ |
|  | Videos |  |  |  |  |  |  |  |  |  | 9.715 | 0.36 | 2.30^*^ |
|  | SNS |  |  |  |  |  |  |  |  |  | 0.517 | 0.01 | 0.12 |
| Statistics of the model | | F = 3.817^*^, R^2^ = .052 | | | F = 14.244^***^, R^2^ = .388,  F Change = 18.496^***^,  R^2^ Change = 0.336 | | | F = 13.692^***^, R^2^ = .417,  F Change = 6.744^*^,  R^2^ Change =0.029 | | | F = 12.656^***^, R^2^ = .511,  F Change = 6.385^***^,  R^2^ Change = 0.074 | | |

*: *P*<.05, **: *P*<.01, ***: *P*<.001, YIAS: Young Internet Addiction Scale Score, SE: Two-Factor Self-Esteem Scale, PHQ-9: The Patient Health Questionnaire 9, K-SPIN: The Korean version of the Social Phobia Inventory, K-ARS: Korean version of the attention deficit hyperactivity disorder scale

Table S3. Hierarchical linear regression analyses with the YIAS score as the dependent variable in 2020

| Independent variables | | Model 1 | | | Model 2 | | | Model 3 | | | Model 4 | | |
| --- | --- | --- | --- | --- | --- | --- | --- | --- | --- | --- | --- | --- | --- |
|  |  | B | Beta | t | B | Beta | t | B | Beta | t | B | Beta | t |
| Demographic factors | Age | -5.28 | -0.20 | -2.28^*^ | 0.11 | 0.00 | 0.06 | 0.06 | 0.00 | 0.04 | 0.58 | 0.02 | 0.31 |
|  | Sex (female) | 0.79 | 0.07 | 0.76 | 0.23 | 0.02 | 0.30 | 0.27 | 0.02 | 0.34 | 0.44 | 0.04 | 0.57 |
| Psychological  Test | SE |  |  |  | 0.06 | 0.05 | 0.77 | 0.06 | 0.05 | 0.78 | 0.10 | 0.09 | 1.28 |
|  | PHQ9 |  |  |  | 0.23 | 0.08 | 1.06 | 0.24 | 0.08 | 1.08 | 0.18 | 0.06 | 0.81 |
|  | KSPIN |  |  |  | -0.13 | -0.07 | -1.04 | -0.13 | -0.07 | -1.05 | -0.12 | -0.06 | -0.95 |
|  | KARS |  |  |  | 0.76 | 0.64 | 7.73^***^ | 0.75 | 0.64 | 7.65^***^ | 0.71 | 0.60 | 7.30^***^ |
| IT use time | Time |  |  |  |  |  |  | -0.20 | -0.02 | -0.26 | -0.05 | 0.00 | -0.06 |
| IT activity | Game |  |  |  |  |  |  |  |  |  | 6.62 | 0.24 | 2.25^*^ |
|  | Videos |  |  |  |  |  |  |  |  |  | 6.72 | 0.24 | 2.28^*^ |
|  | SNS |  |  |  |  |  |  |  |  |  | 0.95 | 0.03 | 0.27 |
| Statistics of the model | | F = 2.910, R^2^ = .045 | | | F = 20.676^***^, R^2^ = .510,  F Change = 28.269^***^,  R^2^ Change = 0.465 | | | F = 17.593^***^, R^2^ = .511,  F Change = 0.066,  R^2^ Change <0.001 | | | F = 14.104^***^, R^2^ = .551,  F Change = 6.385^***^,  R^2^ Change = 0.074 | | |

*: *P*<.05, **: *P*<.01, ***: *P*<.001, YIAS: Young Internet Addiction Scale Score, SE: Two-Factor Self-Esteem Scale, PHQ-9: The Patient Health Questionnaire 9, K-SPIN: The Korean version of the Social Phobia Inventory, K-ARS: Korean version of the attention deficit hyperactivity disorder scale

Table S4. Hierarchical linear regression analyses with the YIAS score as the dependent variable in 2021

| Independent variables | | Model 1 | | | Model 2 | | | Model 3 | | | Model 4 | | |
| --- | --- | --- | --- | --- | --- | --- | --- | --- | --- | --- | --- | --- | --- |
|  |  | B | Beta | t | B | Beta | t | B | Beta | t | B | Beta | t |
| Demographic factors | Age | -4.08 | -0.15 | -1.69 | -3.23 | -0.12 | -1.56 | -3.46 | -0.13 | -1.66 | -3.80 | -0.14 | -1.70 |
|  | Sex (female) | -0.51 | -0.05 | -0.56 | -0.91 | -0.09 | -1.17 | -0.78 | -0.08 | -0.99 | -0.54 | -0.05 | -0.68 |
| Psychological  Test | SE |  |  |  | 0.09 | 0.08 | 0.89 | 0.08 | 0.07 | 0.77 | 0.05 | 0.05 | 0.51 |
|  | PHQ9 |  |  |  | 0.98 | 0.49 | 6.14^***^ | 1.00 | 0.50 | 6.22 | 1.04^***^ | 0.51 | 6.49^***^ |
|  | KSPIN |  |  |  | 0.41 | 0.16 | 1.60 | 0.41 | 0.16 | 1.59 | 0.37 | 0.14 | 1.47 |
|  | KARS |  |  |  | 0.26 | 0.17 | 2.03 | 0.26 | 0.18 | 2.07^*^ | 0.23 | 0.16 | 1.87 |
| IT use time | Time |  |  |  |  |  |  | 0.61 | 0.08 | 1.02 | 0.54 | 0.07 | 0.92 |
| IT activity | Game |  |  |  |  |  |  |  |  |  | 3.56 | 0.12 | 1.00 |
|  | Videos |  |  |  |  |  |  |  |  |  | 7.03 | 0.25 | 2.00^*^ |
|  | SNS |  |  |  |  |  |  |  |  |  | -0.92 | -0.02 | -0.23 |
| Statistics of the model | | F = 1.580, R^2^ = .024 | | | F = 10.106^***^, R^2^ = .330,  F Change = 14.045^***^,  R^2^ Change = 0.306 | | | F = 8.814^***^, R^2^ = .336,  F Change = 1.041,  R^2^ Change =0.006 | | | F = 7.339^***^, R^2^ = .381,  F Change = 2.924^*^,  R^2^ Change = 0.046 | | |

*: *P*<.05, **: *P*<.01, ***: *P*<.001, YIAS: Young Internet Addiction Scale Score, SE: Two-Factor Self-Esteem Scale, PHQ-9: The Patient Health Questionnaire 9, K-SPIN: The Korean version of the Social Phobia Inventory, K-ARS: Korean version of the attention deficit hyperactivity disorder scale

Table S5. Hierarchical linear regression analyses with the YIAS score as the dependent variable in 2022

| Independent variables | | Model 1 | | | Model 2 | | | Model 3 | | | Model 4 | | |
| --- | --- | --- | --- | --- | --- | --- | --- | --- | --- | --- | --- | --- | --- |
|  |  | B | Beta | t | B | Beta | t | B | Beta | t | B | Beta | t |
| Demographic factors | Age | -0.12 | 0.00 | -0.06 | 0.01 | 0.00 | 0.00^*^ | 0.03 | 0.00 | 0.02 | 0.72^*^ | 0.03 | 0.38 |
|  | Sex (female) | 2.91 | 0.16 | 1.93 | 3.21 | 0.18 | 2.52 | 3.21 | 0.18 | 2.51 | 2.28 | 0.13 | 1.78 |
| Psychological  Test | SE |  |  |  | 0.04 | 0.04 | 0.53 | 0.04 | 0.04 | 0.54 | 0.01 | 0.01 | 0.14 |
|  | PHQ9 |  |  |  | 0.64 | 0.29 | 3.15^**^ | 0.64 | 0.29 | 3.13 | 0.69^**^ | 0.31 | 3.50^**^ |
|  | KSPIN |  |  |  | 0.24 | 0.09 | 1.16 | 0.25 | 0.10 | 1.20 | 0.25 | 0.10 | 1.22 |
|  | KARS |  |  |  | 0.41 | 0.32 | 3.35^**^ | 0.40 | 0.31 | 3.22 | 0.33^**^ | 0.25 | 2.67^**^ |
| IT use time | Time |  |  |  |  |  |  | 0.37 | 0.03 | 0.39 | 0.71 | 0.05 | 0.77 |
| IT activity | Game |  |  |  |  |  |  |  |  |  | 10.11 | 0.37 | 3.12^**^ |
|  | Videos |  |  |  |  |  |  |  |  |  | 11.58 | 0.43 | 3.58^***^ |
|  | SNS |  |  |  |  |  |  |  |  |  | 7.86 | 0.25 | 2.33^*^ |
| Statistics of the model | | F = 1.949, R^2^ = .027 | | | F = 11.451^***^, R^2^ = .336,  F Change = 15.790^***^,  R^2^ Change = 0.309 | | | F = 9.776^***^, R^2^ = .302,  F Change = 0.151,  R^2^ Change =0.001 | | | F = 8.706^***^, R^2^ = .352,  F Change = 4.458^**^,  R^2^ Change = 0.061 | | |

*: *P*<.05, **: *P*<.01, ***: *P*<.001, YIAS: Young Internet Addiction Scale Score, SE: Two-Factor Self-Esteem Scale, PHQ-9: The Patient Health Questionnaire 9, K-SPIN: The Korean version of the Social Phobia Inventory, K-ARS: Korean version of the attention deficit hyperactivity disorder scale

Table S6. Hierarchical linear regression analyses with the YIAS score as the dependent variable in 2023

| Independent variables | | Model 1 | | | Model 2 | | | Model 3 | | | Model 4 | | |
| --- | --- | --- | --- | --- | --- | --- | --- | --- | --- | --- | --- | --- | --- |
|  |  | B | Beta | t | B | Beta | t | B | Beta | t | B | Beta | t |
| Demographic factors | Age | -4.88 | -0.16 | -1.95 | -4.10 | -0.14 | -2.81 | -4.11 | -0.14 | -2.78 | -3.63 | -0.12 | -2.35 |
|  | Sex (female) | 2.65 | 0.16 | 1.96 | 1.07 | 0.07 | 1.37 | 1.06 | 0.07 | 1.30^**^ | 0.55 | 0.03 | 0.68^*^ |
| Psychological  Test | SE |  |  |  | 0.06 | 0.05 | 1.03 | 0.06 | 0.05 | 1.02 | 0.07 | 0.06 | 1.13 |
|  | PHQ9 |  |  |  | 1.15 | 0.56 | 10.48^**^ | 1.15 | 0.56 | 10.33^***^ | 1.17 | 0.57 | 10.40^***^ |
|  | KSPIN |  |  |  | -0.49 | -0.11 | -2.23^*^ | -0.49 | -0.11 | -2.21^*^ | -0.36 | -0.08 | -1.64 |
|  | KARS |  |  |  | 0.48 | 0.34 | 5.98^***^ | 0.48 | 0.34 | 5.95^***^ | 0.47 | 0.33 | 5.97^***^ |
| It use time | Time |  |  |  |  |  |  | 0.03 | 0.00 | 0.07 | -0.12 | -0.01 | -0.27 |
| IT activity | Game |  |  |  |  |  |  |  |  |  | 8.74 | 0.24 | 2.55^**^ |
|  | Videos |  |  |  |  |  |  |  |  |  | 7.22 | 0.28 | 3.03^*^ |
|  | SNS |  |  |  |  |  |  |  |  |  | 8.89 | 0.26 | 3.02^**^ |
| Statistics of the model | | F = 4.565^*^, R^2^ = .062 | | | F = 51.865^***^, R^2^ = .701,  F Change = 70.859^***^,  R^2^ Change = 0.638 | | | F = 44.124^***^, R^2^ = .701,  F Change = 0.005,  R^2^ Change <0.001 | | | F = 33.705^***^, R^2^ = .723,  F Change = 3.513^*^,  R^2^ Change = 0.023 | | |

*: *P*<.05, **: *P*<.01, ***: *P*<.001, YIAS: Young Internet Addiction Scale Score, SE: Two-Factor Self-Esteem Scale, PHQ-9: The Patient Health Questionnaire 9, K-SPIN: The Korean version of the Social Phobia Inventory, K-ARS: Korean version of the attention deficit hyperactivity disorder scale
